# Torque expression capacity of 0.018 and 0.022 bracket slots by changing archwire material and cross section

**DOI:** 10.1186/s40510-014-0053-x

**Published:** 2014-09-25

**Authors:** Angela Arreghini, Luca Lombardo, Francesco Mollica, Giuseppe Siciliani

**Affiliations:** Postgraduate School of Orthodontics, University of Ferrara, Via Montebello 31, 44100 Ferrara, Italy; Postgraduate School of Orthodontics, University of Ferrara, Ferrara, Italy; Department of Engineering, University of Ferrara, Via Montebello 31, 44100 Ferrara, Italy

**Keywords:** Torque expression, Slot precision, Play

## Abstract

**Background:**

The aim of this study is to calculate and compare the play and torque expression of 0.018 and 0.022 bracket slots when engaged with archwires of different size, cross section and material.

**Methods:**

Eight orthodontic brackets, two of slot height 0.018 and six of slot height 0.022, from different manufacturers, were measured and fixed to a vertical support. Twenty-four archwires of differing size, cross section and material were selected, measured and tested in each bracket of compatible slot width. Compression testing by Instron dynamometer and geometric calculations enabled us to determine the play angle of each bracket/archwire combination, and the angle at which a clinically efficacious force couple, sufficient for dental movement, is exerted.

**Results:**

All bracket/archwire combinations considered were found to have play angles far above the ideal. This is ascribable to the slots being oversized with respect to the manufacturers' claims. Likewise, some archwires were found to be oversized, while others undersized.

When the same archwire was tested with brackets from different manufacturers, the play and torque expression differed, despite the same nominal dimensions of the slots. When the same bracket was tested with the same size archwires, their construction material was found to influence the torque expression, due to the difference in elastic modulus, but not the wire/slot play.

**Conclusions:**

The dimensional precision of orthodontic brackets and archwires and the rigidity of the latter have a profound influence on the torque expression of pre-angled appliances.

## Background

In the straight-wire technique, brackets are pre-programmed with first-, second- and third-order information, which is expressed thanks to the interplay between the archwire and slot, a function of their respective geometries and sizes. When an undersized archwire is inserted into a bracket slot, the wire can rotate clockwise or anticlockwise. The angle of freedom of the wire within the bracket slot is known as ‘play’, and this increases as the difference in size between the archwire and the slot [1].

Within this range of rotation, no dental movement occurs, so to transmit third-order information to the tooth, the archwire must come into contact with the walls of the slot and then undergo further torsion, generating a force couple through which a moment, or torque, is expressed. In 1982, Burstone stated that a clinically efficacious moment is between 5 and 20 Nmm [2], i.e. no tooth movement occurs under 5 Nmm, and torque exceeding 20 Nmm is likely to damage the periodontal tissues.

Hence, the effective size of the slot is of fundamental importance in orthodontic biomechanics. The earliest edgewise appliances designed by Angle in 1925 [3] featured brackets with slots of height 0.022 inch. In 1930, however, with the introduction of more rigid steel alloys, archwire diameters began to get smaller. This led Steiner, in 1952, to design a bracket with slot 0.018, which were threaded with working archwires of cross section .017 × .025 and full-thickness archwires of .018 × .025 [4]. From the 1970s onwards, first, Andrews then Roth introduced and perfected the straight-wire technique, using working wires of dimensions .019 × .025 and greater thickness wires of .021 × .025 in slot size 0.022 [5]. This marked the start of a divergence between two of the most widespread orthodontic systems: those involving 0.022 inch slots and those relying on 0.018 inch slots.

Over the same period, the archwires also began to evolve. In the 1930s, the first chromium/nickel/steel alloy wires were introduced [6,7], and in the 1950s, the Elgin Watch Company developed chromium/cobalt archwires, whose rigidity increases when heat-treated [8]. In the 1960s, the US Navy created a revolutionary ‘shape-memory’ alloy, Nitinol, which is 20% more elastic than conventional steel and has a much broader field of action. This was followed, in 1980, by the Ormco Corporations launch of beta-titanium (TMA) archwires, made of a formable alloy of elasticity between steel and nickel titanium (NiTi) [9].

The .022 inch system has mechanical advantages in some clinical situations, such as during sliding mechanics when a .019 × .025″ SS archwire is used, nevertheless, .018 inch system seems to be superior in the amount of the couple it is able to express, when a .017 × .025″ SS archwire is engaged [10]. On the other hand, clinical studies on the final outcome of .018 and .022 inch systems did not show any significant difference, as the operator experience seems to be the fundamental parameter [11].

Both the properties of the material from which it is made (elastic modulus and elastic or superelastic behaviour) and the geometry (cross section and relative size to the slot) of an archwire will influence its capacity to express torque [12]. For this reason, our aim was to determine the passive play and the relative torque expression capacity of brackets with 0.018-inch and 0.022-inch slots when threaded with archwires of different size, cross section and material.

The null hypothesis is that there is no difference in the play and in the torque expression when different material and cross section archwires are engaged in bracket with the same slot height.

## Methods

**Figure 1:**
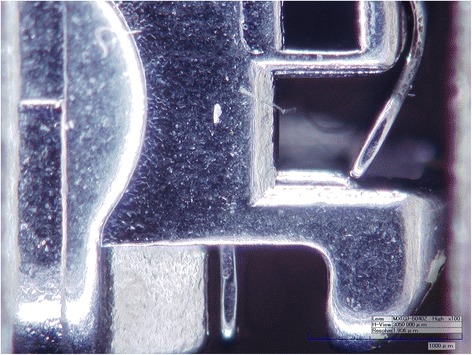
**Nexus bracket photographed under electron microscope.**

Eight different brackets produced by three different manufacturers were selected: two of slot 0.018 inch and six of slot 0.022 inch, of which four were conventional brackets and two self-ligating (Table 1). A clamp was used to fix the vertical position of each bracket, and the slots were photographed at magnification ×100 using a Hirox CT-7700 digital microscope (Hackensack, NJ,USA), whose precision is of the order of tenths of a millimetre (Figure 1). ImageJ software (Cambridge, UK) was then used to measure the height of the slot, i.e. the distance between its occlusal and gingival edges. As brackets feature a certain amount of divergence, the height of each slot was measured at three points: that closest to the base, that on the external surface and that midway between the two. A mean of these three measurements was then compared with the nominal slot height declared by the manufacturer.

**Table 1:** **Brackets selected for the study**

**Bracket**	**Manufacturer**	**Tooth**	**Slot height (inch)**	**Tip**	**Torque**
Victory	3M UNITECK	3.3	.022	3°	0°
SFP	Lancer	3.3	.022	3°	0°
Ovation	GAC Dentsply	4.1	.022	2°	−1°
D.B. Standard	Leone	4.1	.022	0°	0°
Damon	Ormco	3.3	.022	5°	0°
Nexus	Ormco	4.1	.022	0°	0°
STB	Ormco	Lower premolar	.018	0°	0°
Resolve VS 2D	GAC Dentsply	4.1	.018	0°	0°

Twenty-four archwires from two different manufacturers were selected, ten steel, two supertempered steel, nine NiTi and three TMA. Seven of these wires had a square cross section and 17 rectangular (Table 2). The height and width of each archwire was measured three times by the same operator using a Midway digital micrometre (Vogel, Orange City, IA, USA), whose precision is stated to be ±1μm. For each wire tested, the mean and standard deviation of the three measurements was calculated, and via calculation of the standard error of the mean statistical test, the former was compared with the nominal archwire dimensions declared by the manufacturer (*P* < 0.01).

**Table 2:** **Archwires selected for the study**

**Material**	**Cross section**	**Size**	**Type**	**Manufacturer**
SS	Square	.016 × .016	Straight	Ormco
STB SS	Square	.017 × .017	Curved	Ormco
STB SS	Square	.018 × .018	Curved	Ormco
SS	Rectangular	.016 × .022	Straight	Ormco
SS	Rectangular	.017 × .022	Straight	Ormco
SS	Rectangular	.017 × .025	Straight	Ormco
SS	Rectangular	.018 × .022	Straight	Ormco
SS	Rectangular	.018 × .025	Straight	Ormco
SS	Rectangular	.019 × .025	Straight	Ormco
SS	Rectangular	.021 × .025	Straight	Ormco
Supertempered SS	Rectangular	.016 × .022	Curved	GAC DENTSPLY
Supertempered SS	Rectangular	.018 × .022	Curved	GAC DENTSPLY
STB TMA	Square	.0175 × .0175	Curved	Ormco
Damon TMA	Rectangular	.019 × .025	Curved	Ormco
Damon TMA	Rectangular	.021 × .025	Curved	Ormco
STB CuNiTi	Square	.016 × .016	Curved	Ormco
STB CuNiTi	Square	.017 × .017	Curved	Ormco
STB CuNiTi	Square	.018 × .018	Curved	Ormco
Smartclip Nitinol SuperElastic Dimpled	Rectangular	.017 × .025	Curved	3M Unitek
Damon CuNiTi	Rectangular	.018 × .025	Curved	Ormco
Nitinol SuperElastic	Rectangular	.019 × .025	Curved	3M Unitek
Nitinol Heat-Activated	Rectangular	.019 × .025	Curved	3M Unitek
Smartclip Nitinol Hybrid	Rectangular	.019 × .025	Curved	3M Unitek
Nitinol Heat-Activated	Rectangular	.021 × .025	Curved	3M Unitek

**Figure 2:**
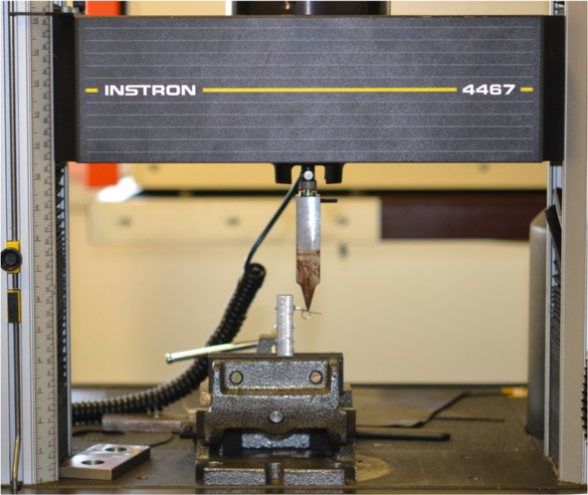
**Torque testing using an Instron 4467 dynamometer.**

**Figure 3:**
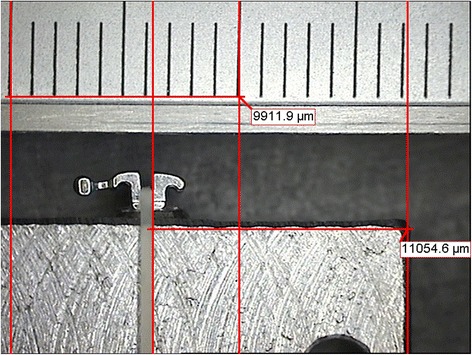
**Measuring the distance between bracket slot and top of stand.**

To calculate the real-world play between the various archwires and bracket slots, load-deflection testing was performed using an Instron 4467 dynamometer (Instron, Norwood, MA, USA) featuring a 100 N load cell and a testing strip (tip radius of curvature 1 mm). The vertical displacement of the strip was transformed into torque of the archwire in the brackets (Figure 2). The brackets were welded to a metal stand with their slots perfectly parallel to the base, under the guidance of a viewfinder at a ×5 magnification, so as to cancel out their tip and torque values. The stand, with brackets attached, was then photographed using a Leica MZ6 optical microscope (Solms, Germany), and Aquinto A4I Docu software (Frankfurt, Germany) was used to verify the effective parallelism of the bracket to the base of support, and to measure the distance between the top of the stand and the upper edge of each slot, to define its position with as much precision as possible (Figure 3).

**Figure 4:**
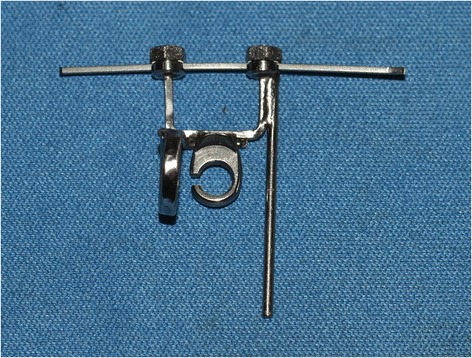
**Torquing key with archwire engaged.**

Each archwire was then engaged in a ‘torquing key’, a type of pliers purposely designed to clamp the wire at two points, 6 mm apart. The device also features a rod on the same plane as the orthodontic archwire, perpendicular to the plier clamps, marked at a fixed distance of 11.15 mm from the same (Figure 4). The purpose of this key was to hold the archwire fast while allowing it to torque upon contact between the Instron testing strip and the post, perpendicular to the plier clamps, maintaining the bar and archwire on the same plane.

**Figure 5:**
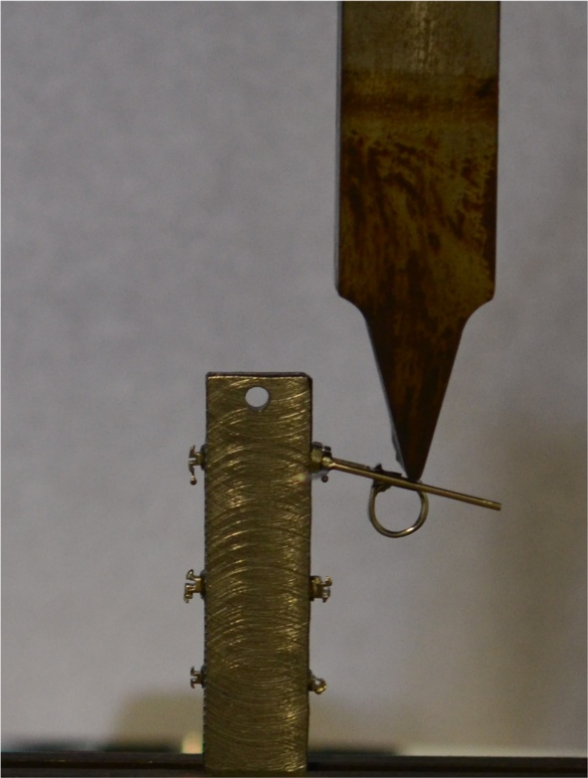
**Torque testing.**

**Figure 6:**
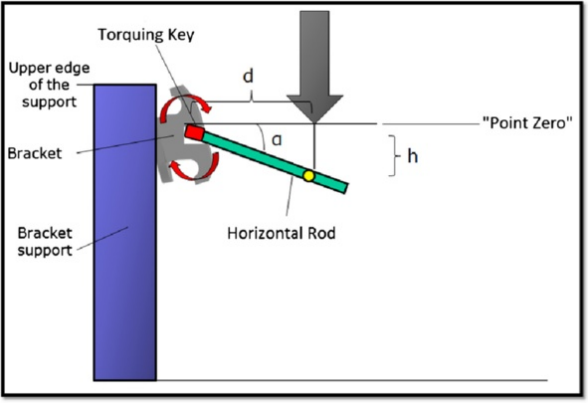
**Schematic of torque testing.**

The bracket stand was fixed to the base of the dynamometer at a horizontal distance of 11.15 mm between bracket and testing strip. The testing strip was lowered until it came into contact with the top edge of the stand and then moved to the exact height of the slot (point zero). The archwire, still clamped in the torquing key, was engaged in the slot and fixed in place with an elastic ligature (conventional brackets), or by closing the active clip (self-ligating). Engaging the archwire, the torquing key rod was spontaneously lowered with respect to the horizontal plane due to ‘passive’ play. Lowering the Instron testing strip from point zero to the point of first contact with the torquing key (h) enables the distance (d) (Figures 5 and 6) and, therefore, the play angle (*α*) to be determined. Using the Instron machine to lower the testing strip still further, a load is exerted on the key. The archwire rotates within the slot and torque is exerted.

**Figure 7:**
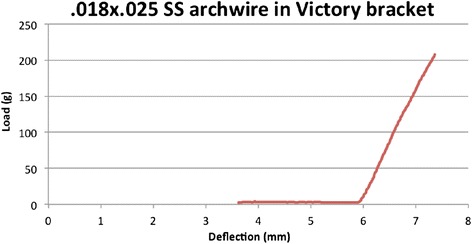
**Load/deflection curve, steel archwire.**

**Figure 8:**
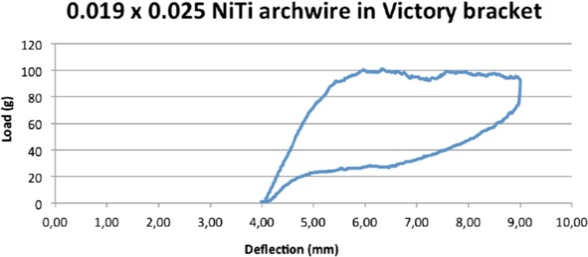
**Load/deflection curve, NiTi archwire.**

From the results of this test, we plotted a load/deflection curve for each experimental bracket/wire combination. For the elastic archwires (steel, supertempered steel and TMA), we considered the loading curve, while in the case of the super-elastic archwires, we considered the curve generated during the dynamometer strip return phase (Figures 7 and 8). Knowing the distance *d* between strip and bracket, and fixing the torque *K* at 5 and 20 Nmm, we were able to identify the loaf *F* at which the archwire expresses moments of 5 and 20 Nmm (the extremes of the clinically efficacious range), by means of the formula *F = K / d*, so the deflection of the strip and the corresponding angles.

Each compatible archwire was tested three times in each bracket, and in each test, the passive play angle, the torque angle at 5 Nmm and the torque angle at 20 Nmm was identified. This data was analysed as follows:

- First, using previously described calculations [13], the ideal play for each archwire in each slot was identified, i.e. the angle of engagement that would result if the real-world dimensions of the slot and archwire matched those declared by their respective manufacturers, and if the archwire had 90° edge bevels.
- Second, this figure was compared with the real-world play we measured earlier, using the standard error of the mean calculation statistical test to determine whether any differences were significant (*P* < 0.01).
- Third, for each single archwire in each compatible bracket of nominally identical slot dimensions, the clinical significant torque angles were calculated.

## Results

Table 3 shows the measured slot heights of each tested bracket in both inches and millimetres, and their percentage variation from those declared by their respective manufacturers. This data shows that all brackets tested featured oversized slots, with the Lancer SFP (Vista, CA, USA) being the most faithful to its stated measurements (+0.56%), and the Ormco Victory (Orange, CA, USA) the least (+11.16%).

**Table 3:** **Analysis of slot dimensions**

	**Stated size**	**Measured size (mm)**	**SD**	**Measured size (inch)**	**SD**	**% Increase**
Victory	0.56 mm	0.6211	0.0227	0.0245	0.0009	11.16
	.022 inch					
SFP	0.56 mm	0.5619	0.0250	0.0221	0.0010	0.56
	.022 inch					
Leone	0.56 mm	0.6191	0.0259	0.02437	0.0010	10.79
	.022 inch					
Ovation	0.56 mm	0.5896	0.0082	0.0232	0.0003	5.51
	.022 inch					
Damon	0.56 mm	0.6033	0.0199	0.0238	0.0008	7.96
	.022 inch					
Nexus	0.56 mm	0.5924	0.0059	0.0233	0.0002	6.01
	.022 inch					
STB	0.46 mm	0.4772	0.0174	0.0188	0.0007	4.37
	.018 inch					
GAC BIDI	0.46 mm	0.4629	0.0085	0.0182	0.0003	1.25
	.018 inch					

Table 4 shows the measured heights and widths of each tested archwire, their percentage variation with those declared by their respective manufacturers and the statistical significance of any differences. Roughly half of the archwires tested were found to be oversized, and the remainder undersized, in a range spanning −5.41% to +2.44%.

**Table 4:** **Analysis of archwire dimensions**

**Archwire**	**Parameter**	**Stated size**	**Measured size (mm)**	**SD**	**Measured size (inch)**	**SD**	**% difference**	**Significant difference (** ***P*** **< 0.01)**
SS .016 × .016	Height	0.41	0.401	0.008	0.0158	0.0003	−1.25	NS
	Width	0.41	0.393	0.002	0.0155	0.0001	−3.22	S
SS .016 × .022	Height	0.41	0.411	0.003	0.0161	0.0001	1.13	NS
	Width	0.56	0.558	0.003	0.0220	0.0001	−0.14	NS
SS .017 × .017	Height	0.43	0.434	0.002	0.0170	0.00007	0.51	S
	Width	0.43	0.435	0.001	0.0171	0.0000	0.74	S
SS .017 × .022	Height	0.43	0.430	0.002	0.0169	0.00008	−0.34	NS
	Width	0.56	0.552	0.001	0.0217	0.0000	−1.28	S
SS .017 × .025	Height	0.43	0.428	0.002	0.0168	0.00006	−0.96	NS
	Width	0.64	0.635	0.003	0.0250	0.0001	0.00	S
SS .018 × .018	Height	0.46	0.458	0.001	0.0180	0.00002	0.25	S
	Width	0.46	0.456	0.003	0.0180	0.0001	−0.19	NS
SS .018 × .022	Height	0.46	0.438	0.003	0.0172	0.00012	−4.20	S
	Width	0.56	0.554	0.002	0.0218	0.0001	−0.80	S
SS .018 × .025	Height	0.46	0.451	0.001	0.0177	0.00005	−1.43	S
	Width	0.64	0.636	0.003	0.0250	0.0001	0.16	NS
SS .019 × .025	Height	0.48	0.488	0.001	0.0192	0.00004	1.12	S
	Width	0.64	0.636	0.002	0.0250	0.0001	0.10	S
SS .021 × .025	Height	0.53	0.539	0.002	0.0212	0.00006	0.99	S
	Width	0.64	0.636	0.001	0.0250	0.0000	0.16	S
SS ST .016 × .022	Height	0.41	0.404	0.001	0.0159	0.0003	−0.59	S
	Width	0.56	0.556	0.002	0.0218	0.0008	−0.56	S
SS ST .018 × .022	Height	0.46	0.445	0.001	0.0175	0.0006	−2.74	S
	Width	0.56	0.560	0.001	0.022	0,0006	0.27	NS
TMA .0175 × .0175	Height	0.44	0.447	0.001	0.0176	0.00004	0.56	S
	Width	0.44	0.444	0.002	0.0175	0.0001	−0.11	S
TMA .019 × .025	Height	0.48	0.488	0.002	0.0192	0.00006	1.05	S
	Width	0.64	0.643	0.002	0.0253	0.0001	1.26	S
TMA .021 × .025	Height	0.53	0.539	0.002	0.0212	0.00006	1.11	S
	Width	0.64	0.623	0.003	0.0245	0.0001	−1.89	S
CuNiTi .016 × .016	Height	0.41	0.415	0.001	0.0163	0.00004	2.12	S
	Width	0.41	0.416	0.003	0.0164	0.0001	2.44	S
CuNiTi .017 × .017	Height	0.43	0.425	0.001	0.0167	0.00002	−1.65	S
	Width	0.43	0.425	0.004	0.0167	0.0002	−1.65	NS
Nitinol .017 × .025	Height	0.43	0.432	0.001	0.0170	0.00004	0.05	S
	Width	0.64	0.639	0.001	0.0252	0.0000	0.63	NS
CuNiTi .018 × .018	Height	0.46	0.449	0.001	0.0176	0.00005	−1.87	S
	Width	0.46	0.449	0.001	0.0177	0.0000	−1.72	S
CuNiTi .018 × .025	Height	0.46	0.456	0.001	0.0179	0.00004	−0.26	S
	Width	0.64	0.628	0.001	0.0247	0.0000	−1.05	S
Nitinol Heat .019 × .025	Height	0.48	0.477	0.002	0.0187	0.00006	−1.09	S
	Width	0.64	0.635	0.001	0.0250	0.0000	−0.05	S
Nitinol SE .019 × .025	Height	0.48	0.480	0.001	0.0189	0.00004	−0.54	NS
	Width	0.64	0.601	0.058	0.0236	0.0023	−5.41	NS
Nitinol Hybrid .019 × .025	Height	0.48	0.485	0.004	0.0191	0.00016	0.57	NS
	Width	0.64	0.625	0.004	0.0246	0.00016	−1.57	S
Nitinol Heat .021 × .025	Height	0.53	0.528	0.003	0.0207	0.00012	−1.08	NS
	Width	0.64	0.633	0.002	0.0249	0.0001	−0.37	S

Tables 5, 6, 7, 8, 9 show the ideal archwire/slot play, the measured archwire/slot play, the standard deviation, the standard error of the mean and the statistical significance (*P* < 0.01). All archwire/bracket combinations exhibited a significantly greater measured play angle than the ideal. What is more, several of the archwires tested rotated within the slots, despite their rectangular shape.

**Table 5:** **A comparison of real and ideal play of 0.022 bracket and SS archwire combinations**

	**Ideal play (°)**	**Bracket**	**Engagement angle (°)**	**SD**	**Mean standard error**	**Significant difference**
SS.0 16 × .022	17.95	Victory	Rotates	-	-	-
		SFP	37.94	0.59	0.34	S
		Ovation	36.55	1.23	0.71	S
		D.B. Leone	Rotates	-	-	-
		Damon	39.63	0.39	0.22	S
		Nexus	Rotates	-	-	-
SS .017 × .022	14.61	Victory	36.34	2.07	1.20	S
		SFP	27.75	1.10	0.63	S
		Ovation	26.56	0.46	0.27	S
		D.B. Leone	30.51	2.44	1.41	S
		Damon	29.54	0.98	0.57	S
		Nexus	Rotates	-	-	-
SS .017 × .025	12.48	Victory	28.45	1.42	0.82	S
		SFP	20.43	0.59	0.34	S
		Ovation	21.53	0.40	0.23	S
		D.B. Leone	22.74	0.15	0.09	S
		Damon	21.59	0.67	0.38	S
		Nexus	40.75	1.61	0.93	S
SS .018 × .022	11.42	Victory	28.72	0.12	0.07	S
		SFP	21.40	0.23	0.13	S
		Ovation	20.91	0.11	0.07	S
		D.B. Leone	23.38	0.67	0.39	S
		Damon	19.86	2.24	1.29	S
		Nexus	40.43	0.03	0.02	S
SS .018 × .025	9.82	Victory	26.88	0.23	0.13	S
		SFP	17.56	0.27	0.16	S
		Ovation	18.20	0.31	0.18	S
		D.B. Leone	21.64	0.42	0.24	S
		Damon	19.85	1.15	0.66	S
		Nexus	35.42	0.70	0.41	S
SS .019 × .025	7.24	Victory	18.41	1.87	1.08	S
		SFP	14.36	0.10	0.06	S
		Ovation	13.80	0.12	0.07	S
		D.B. Leone	16.08	0.22	0.13	S
		Damon	14.84	0.23	0.13	S
		Nexus	28.68	0.48	0.27	S
SS .021 × .025	2.33	Victory	13.65	0.08	0.05	S
		SFP	5.60	0.11	0.06	S
		Ovation	5.49	0.10	0.06	S
		D.B. Leone	9.00	0.41	0.23	S
		Damon	5.12	0.10	0.06	S
		Nexus	20.99	0.27	0.16	S

**Table 6:** **A comparison of real and ideal play of 0.022 bracket and supertempered steel archwire combination**

	**Ideal play (°)**	**Bracket**	**Engagement angle (°)**	**SD**	**Mean standard error**	**Significant difference**
ST SS .016 × .022	17.95	Victory	35.40	0.32	0.19	S
		SFP	32.55	0.95	0.55	S
		Ovation	31.37	0.17	0.10	S
		D.B. Leone	Rotates	-	-	-
		Damon	26.09	0.77	0.45	S
		Nexus	Rotates	-	-	-
ST SS .018 × .022	11.42	Victory	29.43	1.00	0.58	S
		SFP	25.66	0.54	0.31	S
		Ovation	23.74	0.34	0.20	S
		D.B. Leone	29.34	1.95	1.13	S
		Damon	18.80	0.44	0.25	S
		Nexus	Rotates	-	-	-

**Table 7:** **A comparison of real and ideal play of 0.022 bracket and TMA archwire combinations**

	**Ideal play (°)**	**Bracket**	**Engagement angle (°)**	**SD**	**Mean standard error**	**Significant difference**
TMA .019 × .025	7.24	Victory	22.30	0.23	0.13	S
		SFP	15.30	0.33	0.19	S
		Ovation	15.86	0.24	0.14	S
		D.B. Leone	18.46	0.35	0.20	S
		Damon	15.41	0.55	0.32	S
		Nexus	34.48	2.18	1.26	S
TMA .021 × .025	2.33	Victory	12.94	0.11	0.06	S
		SFP	5.66	0.24	0.14	S
		Ovation	6.08	0.13	0.07	S
		D.B. Leone	9.38	0.35	0.20	S
		Damon	6.09	0.59	0.34	S
		Nexus	14.51	2.51	1.45	S

**Table 8:** **A comparison of real and ideal play of 0.022 bracket and NiTi archwire combinations**

	**Ideal play (°)**	**Bracket**	**Engagement angle (°)**	**SD**	**Mean standard error**	**Significant difference**
Nitinol .017 × .025	12.48	Victory	27.13	1.13	0.66	S
		SFP	21.18	0.75	0.43	S
		Ovation	21.29	1.03	0.59	S
		D.B. Leone	23.95	0.45	0.26	S
		Damon	20.96	0.89	0.51	S
		Nexus	39.00	1.12	0.64	S
CuNiTi .018 × .025	9.82	Victory	32.35	0.96	0.56	S
		SFP	30.13	0.40	0.23	S
		Ovation	23.35	0.70	0.40	S
		D.B. Leone	30.79	0.17	0.10	S
		Damon	26.60	1.98	1.14	S
		Nexus	48.97	0.40	0.23	S
Nitinol .019 × .025 Hybrid	7.24	Victory	28.16	1.05	0.60	S
		SFP	19.17	1.05	0.61	S
		Ovation	19.20	0.56	0.32	S
		D.B. Leone	36.51	3.31	1.91	S
		Damon	21.46	0.96	0.56	S
		Nexus	42.59	0.14	0.08	S
Nitinol Superelastic .019 × 025	7.24	Victory	19.66	0.75	0.44	S
		SFP	15.64	0.48	0.28	S
		Ovation	14.96	0.22	0.13	S
		D.B. Leone	28.30	2.82	1.63	S
		Damon	16.21	0.49	0.28	S
		Nexus	26.42	0.46	0.26	S
Nitinol .019 × .025 Heat	7.24	Victory	20.64	0.43	0.25	S
		SFP	15.91	0.36	0.21	S
		Ovation	13.47	1.52	0.88	S
		D.B. Leone	31.09	1.33	0.77	S
		Damon	18.36	0.55	0.32	S
		Nexus	27.79	0.39	0.23	S
Nitinol .021 × .025 Heat	2.33	Victory	12.42	1.47	0.85	S
		SFP	8.27	0.48	0.28	S
		Ovation	7.46	0.27	0.15	S
		D.B. Leone	15.23	0.03	0.02	S
		Damon	10.66	3.39	1.96	S
		Nexus	17.50	1.88	1.08	S

**Table 9:** **A comparison of real and ideal play of 0.018 brackets**

	**Ideal play (°)**	**Bracket**	**Engagement angle (°)**	**SD**	**Mean standard error**	**Significant difference**
SS .016 × .016	15.40	STB	18.63	1.42	0.82	S
		2D	16.55	0.74	0.43	S
SS .017 × .017	6.96	STB	8.81	0.27	0.16	S
		2D	7.20	0.19	0.11	S
SS .018 × .018	0.00	STB	5.77	0.16	0.09	S
		2D	2.36	0.19	0.11	S
SS .016 × .022	10.80	STB	14.64	0.03	0.02	S
		2D	12.09	0.47	0.27	S
SS .017 × .022	5.31	STB	7.42	0.03	0.02	S
		2D	6.01	0.19	0.11	S
SS .017 × .025	4.65	STB	6.54	0.05	0.03	S
		2D	4.84	0.08	0.05	S
SS .018 × .022	0.00	STB	4.86	0.12	0.07	S
		2D	1.23	0.19	0.11	S
SS .018 × .025	0.00	STB	4.17	0.16	0.09	S
		2D	2.26	0.09	0.05	S
ST SS .016 × .022	10.80	STB	11.30	0.06	0.03	S
		2D	11.30	0.40	0.23	S
ST SS .018 × .022	0.00	STB	10.82	0.13	0.08	S
		2D	4.98	0.14	0.08	S
TMA .0175 × .0175	3.32	STB	9.59	0.87	0.50	S
		2D	6.71	0.69	0.40	S
CuNiTi .016 × .016	15.40	STB	20.83	0.25	0.14	S
		2D	18.59	0.88	0.51	S
CuNiTi .017 × .017	6.96	STB	16.36	0.58	0.33	S
		2D	16.40	0.76	0.44	S
CuNiTi .018 × .018	0.00	STB	8.37	0.46	0.26	S
		2D	5.47	0.74	0.43	S
Nitinol .017 × .025	4.65	STB	7.86	0.18	0.10	S
		2D	5.94	0.15	0.09	S
NiTi .018 × .025	0.00	STB	14.95	0.27	0.15	S
		2D	3.10	0.24	0.14	S

Tables 10, 11, 12, 13, 14 show the angles at which the archwires expressed a clinically significant couple (5 and 20 Nmm). For archwire/bracket combinations that failed to generate a torque of 20 Nmm, the maximum value reached is reported. The final column of the tables shows the clinically significant torque interval for each combination, i.e. how many degrees of torque need to be applied to reach the maximum 20 Nmm couple from the minimum 5 Nmm. This torque is expressed in a single direction, either clockwise or anticlockwise.

**Table 10:** **Torque expression, 0.022 brackets and conventional steel archwires**

	**Bracket**	**5 Nmm angle (°)**	**SD**	**20 Nmm angle (°)**	**SD**	**Maximum moment (Nmm)**	**SD**	**Clinically significant torque (°)**
SS .016 × .022	Victory	-	-	-	-	-	-	-
	SFP	44.70	1.75	-	-	6.76	0.64	-
	Ovation	41.07	1.53	-	-	13.71	0.49	-
	D.B. Leone	-	-	-	-	-	-	-
	Damon	-	-	-	-	3.65	0.21	-
	Nexus	-	-	-	-	-	-	-
SS .017 × .022	Victory	42.83	5.08	-	-	10.07	4.25	-
	SFP	34.15	1.69	-	-	17.67	0.56	-
	Ovation	29.32	0.80	39.39	0.31	-	-	10.07
	D.B. Leone	34.29	3.81	-	-	14.23	2.04	-
	Damon	34.60	1.71	-	-	11.84	0.77	-
	Nexus	-	-	-	-	-	-	-
SS .017 × .025	Victory	29.34	0.78	34.49	0.50	-	-	5.15
	SFP	22.44	0.71	28.95	0.22	-	-	6.50
	Ovation	22.75	0.14	25.41	0.05	-	-	2.66
	D.B. Leone	24.55	0.41	27.99	0.48	-	-	3.44
	Damon	23.19	0.74	30.32	5.44	-	-	7.14
	Nexus	45.07	2.11	-	-	15.01	0.87	-
SS .018 × .022	Victory	31.15	0.26	37.38	0.48	-	-	6.23
	SFP	23.64	0.40	28.14	0.17	-	-	4.51
	Ovation	22.40	0.24	25.00	0.17	-	-	2.60
	D.B. Leone	25.47	1.15	30.46	0.83	-	-	4.98
	Damon	23.37	0.55	32.30	0.50	-	-	8.92
	Nexus	45.46	2.42	-	-	10.21	0.56	-
SS .018 × .025	Victory	28.24	0.25	32.54	0.08	-	-	4.30
	SFP	19.41	0.43	23.65	0.16	-	-	4.24
	Ovation	19.40	0.47	24.71	0.15	-	-	5.31
	D.B. Leone	23.02	0.58	27.15	0.63	-	-	4.13
	Damon	21.39	1.13	29.50	1.51	-	-	8.11
	Nexus	38.21	1.37	-	-	19.09	0.72	-
SS .019 × .025	Victory	21.64	1.85	23.14	0.05	-	-	1.50
	SFP	15.86	0.15	18.86	0.03	-	-	3.00
	Ovation	14.88	0.14	18.11	0.12	-	-	3.23
	D.B. Leone	17.37	0.10	20.71	0.09	-	-	3.34
	Damon	16.08	0.39	23.07	0.44	-	-	6.99
	Nexus	30.43	0.50	35.66	0.22	-	-	5.23
SS .021 × .025	Victory	14.52	0.09	16.90	0.05	-	-	2.38
	SFP	7,.6	0.13	9.94	0.06	-	-	2.88
	Ovation	6.33	0.16	8.88	0.17	-	-	2.54
	D.B. Leone	11.40	0.55	18.68	0.44	-	-	7.28
	Damon	6.06	0.13	8.53	0.13	-	-	2.47
	Nexus	28.31	0.32	37.60	0.18	-	-	9.29

**Table 11:** **Torque expression, 0.022 brackets and supertempered steel archwires**

	**Bracket**	**5 Nmm angle (°)**	**SD**	**20 Nmm angle (°)**	**SD**	**Maximum moment (Nmm)**	**SD**	**Clinically significant torque (°)**
ST SS .016 × .022	Victory	40.89	2.17	-	-	12.81	0.00	-
	SFP	37.10	1.70	-	-	16.67	0.57	-
	Ovation	34.57	0.68	47.21	0.15	-	-	12.64
	D.B. Leone	-	-	-	-	-	-	-
	Damon	29.37	0.77	42.26	3.14	-	-	12.89
	Nexus	-	-	-	-	-	-	-
ST SS .018 × .022	Victory	32.11	1.64	-	-	12.81	0.00	-
	SFP	28.83	1.03	37.10	0.67	-	-	8.27
	Ovation	25.76	0.64	34.34	0.16	-	-	8.59
	D.B. Leone	31.91	2.74	-	-	18.46	0.90	-
	Damon	21.09	0.49	27.16	0.58	-	-	6.07
	Nexus	-	-	-	-	-	-	-

**Table 12:** **Torque expression, 0.022 brackets and TMA archwires**

	**Bracket**	**5 Nmm angle (°)**	**SD**	**20 Nmm angle (°)**	**SD**	**Maximum moment (Nmm)**	**SD**	**Clinically significant torque (°)**
TMA .019 × .025	Victory	23.82	0.34	30.51	0.16	-	-	6.70
	SFP	17.53	0.29	22.59	0.09	-	-	5.06
	Ovation	17.80	0.47	26.45	0.14	-	-	8.65
	D.B. Leone	20.38	0.34	26.98	0.17	-	-	6.60
	Damon	17.03	0.58	23.02	0.40	-	-	5.99
	Nexus	39.64	2.35	-	-	15.12	0.68	-
TMA .021 × .025	Victory	14.03	0.21	17.78	0.03	-	-	3.75
	SFP	7.09	0.20	11.67	0.13	-	-	4.58
	Ovation	7.55	0.13	13.36	0.07	-	-	5.81
	D.B. Leone	10.82	0.26	15.47	0.12	-	-	4.65
	Damon	8.00	0.33	14.00	0.20	-	-	6.00
	Nexus	17.81	2.66	-	-	16.83	1.02	-

**Table 13:** **Torque expression, 0.022 brackets and NiTi archwires**

	**Bracket**	**5 Nmm angle (°)**	**SD**	**20 Nmm angle (°)**	**SD**	**Maximum moment (Nmm)**	**SD**
Nitinol .017 × .025	Victory	34.30	0.29	-	-	9.37	0.32
	SFP	27.06	0.06	-	-	14.42	0.23
	Ovation	28.53	0.13	-	-	12.52	-
	D.B. Leone	31.94	0.09	-	-	12.85	-
	Damon	27.91	0.13	-	-	10.63	0.13
	Nexus	54.79	0.08	-	-	5.24	0.19
CuNiTi .018 × .025	Victory	-	-	-	-	3.67	0.11
	SFP	41.10	0.29	-	-	5.72	0.17
	Ovation	36.15	0.17	-	-	7.84	-
	D.B. Leone	45.84	0.17	-	-	5.98	-
	Damon	-	-	-	-	4.51	0.13
	Nexus	-	-	-	-	1.52	0.23
Nitinol .019 × .025 Hybrid	Victory	39.52	0.27	-	-	8.52	0.17
	SFP	29.54	0.33	-	-	14.30	0.39
	Ovation	30.78	0.12	-	-	11.66	-
	D.B. Leone	-	-	-	-	4.79	-
	Damon	27.84	0.06	-	-	10.81	0.29
	Nexus	-	-	-	-	2.53	0.72
Nitinol Superelastic .019 × .025	Victory	35.16	0.02	-	-	8.09	0.17
	SFP	26.01	0.86	-	-	11.89	0.17
	Ovation	21.55	0.46	-	-	11.81	-
	D.B. Leone	40.83	0.99	-	-	7.95	-
	Damon	36.31	0.48	-	-	8.30	0.66
	Nexus	44.48	0.21	-	-	7.39	0.28
Nitinol .019 × .025 Heat	Victory	34.06	3.55	-	-	7.80	0.00
	SFP	28.38	0.12	-	-	11.29	0.17
	Ovation	22.32	0.13	-	-	10.88	-
	D.B. Leone	41.70	0.55	-	-	7.91	-
	Damon	31.27	0.07	-	-	7.46	0.17
	Nexus	40.00	2.25	-	-	5.79	0.11
Nitinol .021 × .025 Heat	Victory	28.98	2.20	-	-	7.31	0.96
	SFP	18.55	0.21	-	-	15.42	0.56
	Ovation	11.76	0.23	-	-	13.52	-
	D.B. Leone	21.55	0.20	-	-	15.34	-
	Damon	27.70	0.12	-	-	10.81	0.50
	Nexus	29.74	0.14	-	-	9.73	0.13

**Table 14:** **Torque expression, 0.018 brackets**

	**Bracket**	**5 Nmm angle (°)**	**SD**	**20 Nmm angle (°)**	**SD**	**Maximum moment (Nmm)**	**SD**	**Clinically significant torque (°)**
SS .016 × .016	STB	23.66	1.91	-	-	19.13	0.29	-
	2D	23.59	1.17	42.63	0.96	-	-	19.04
SS .017 × .017	STB	10.72	0.21	16.44	0.17	-	-	5.72
	2D	9.51	0.31	17.25	0.16	-	-	7.74
SS .018 × .018	STB	7.24	0.14	11.70	0.10	-	-	4.46
	2D	4.45	0.11	10.49	0.10	-	-	6.04
SS .016 × .022	STB	15.76	0.36	20.25	0.44	-	-	4.49
	2D	13.88	0.47	20.81	0.29	-	-	6.92
SS .017 × .022	STB	8.54	0.13	12.24	0.08	-	-	3.70
	2D	7.47	0.09	11.52	0.00	-	-	4.05
SS .017 × .025	STB	7.42	0.03	10.13	0.00	-	-	2.71
	2D	6.42	0.08	11.34	0.06	-	-	4.92
SS .018 × .022	STB	5.71	0.06	8.51	0.00	-	-	2.80
	2D	3.44	0.40	8.02	0.48	-	-	4.58
SS .018 × .025	STB	5.04	0.24	7.90	0.00	-	-	2.86
	2D	3.95	0.05	7.75	0.06	-	-	3.80
ST SS .016 × .022	STB	12.99	0.18	17.99	0.05	-	-	5.00
	2D	12.68	0.43	17.80	0.16	-	-	5.12
ST SS .018 × .022	STB	12.70	0.13	17.64	0.03	-	-	4.95
	2D	6.10	0.09	9.76	0.03	-	-	3.66
TMA .0175 × .0175	STB	12.94	1.09	26.34	0.66	-	-	13.40
	2D	10.76	0.71	-	-	17.57	1.59	
CuNiTi .016 × .016	STB	-	-	-	-	2.58	0.06	-
	2D	-	-	-	-	3.75	0.06	-
CuNiTi .017 × .017	STB	-	-	-	-	3.17	0.06	-
	2D	-	-	-	-	4.12	0.19	-
CuNiTi .018 × .018	STB	-	-	-	-	4.69	0.06	-
	2D	9.58	0.10	18.33	0.08	-	-	8.75
Nitinol .017 × .025	STB	12.48	0.16	-	-	15.52	0.50	-
	2D	12.66	0.10	-	-	16.75	0.23	-
NiTi .018 × .025	STB	29.63	0.38	-	-	6.52	0.00	-
	2D	12.27	0.23	-	-	10.14	0.11	-

For each archwire tested, the play and torque angles in nominally identical bracket slots were different, in some cases by as much as 100%. The bracket with the largest difference between real and ideal play angles was the Nexus (Valencia, CA, USA). When archwires of nominally identical dimensions but different construction material were compared, it was found that the torque expression interval was invariably greater in NiTi with respect to steel wires, with the TMA wires exhibiting intermediate values.

## Discussion

The play between the archwire and bracket slot is of fundamental importance in clinical orthodontics, as it indicates how many degrees the archwire must be rotated within the bracket before its edges come into contact with the slot walls, enabling it thus to transmit third-order information to the tooth. The degree of play depends entirely on geometric parameters, namely the real slot height, the dimensions of the archwire and the bevelling of its edge. However, on the products on the market, these do not always conform to the measurements declared by the manufacturer. Like other authors, we found that all slots were oversized with respect to the stated dimensions, ranging from +0.56% to +11.16%. This is similar to findings reported by Cash et al. [14], for example, who, however, found that Victory brackets featured slots 6% greater than their purported height, and Damon (Ormco, Orange, CA, USA) bracket slots the difference was as much as 17%, while we measured respective size differences of 11% and 8% for the same bracket types. That being said, both of these brackets feature heavily bevelled edges, which can make it difficult for the operator to perform measurements on photographs yielded by a microscope.

The oversized slot dimension involves both vestibular and lingual brackets. In their study, Demling et al. found that the lingual brackets from any technique are oversized with respect to the ideal dimensions, in a range comparable to that recorded in our research. The torque control is an issue that concerns both the vestibular and the lingual orthodontics [15].

Not only the brackets but also the archwires differed considerably from their stated dimensions. This finding confirms that of reported by both Meling and Ødegaard [1] and Rucker and Cusy [16]. The variability in archwire size that we identified ranged between −5.41% and +2.44%. If the measurements pertaining to the two archwires at the extremes of the scale are discounted, this still leaves 22 out of the 24 falling between the range −3.22% and +2.74%. This interval is similar to that reported by Rucker and Cusy [16], who found a size variation range of −3.2% to +3, 1% in round and rectangular NiTi and SS archwires.

These imprecisions in manufacture inevitably affect the play between the archwire and slot and, therefore, the torque expression capacity of the appliance.

It is, however, difficult to calculate the real archwire/slot play and torque expression, which needs to take into account all the dimensional parameters, and results do not readily lend themselves to generalizations due to the great variability in archwire and bracket slot dimensions between manufacturers, even if they are nominally the same. Indeed, Huang et al. [17] used finite elements analysis to evaluate the play and torque expression of pre-angled appliances, but failed to consider the bevelled edges of the archwires, meaning that those results have little bearing in real-world scenarios. Badawi et al. [18] also set out to measure the archwire/slot play and torque angles, using a dynamometer that engaged the ends of the archwire and progressively rotated it in the slot. We also used a dynamometer, but adopted a torquing key, as proposed by Flores et al. [19,20] to help us measure the torque and play. This approach has the advantage of taking all geometric factors of the archwire and slot into consideration, thereby providing authentic values for play and torque. With this approach, we found that for all archwire/bracket combinations tested, the real play was always greater than the ideal. Indeed, we found that, in reality, the combination SS .019 × .025 wire/0.022 bracket combination had a play between 2.2 and 3.2 greater with respect to the ideal, which is similar to that reported by Badawi et al. [18].

Both play and torque are significantly influenced by bracket and archwire features. In contrast, the material used to make the archwire does not significantly affect the play, although it is a decisive factor in terms of torque. Archambault et al. [21] found that at the same level of torque, a .019 × .025 SS archwire expresses a couple 1.5 to 2 times greater than a TMA wire of the same dimensions and 2.5 to 3 times greater than the NiTi version. These were similar to our results, which also showed that steel archwires express torque at much smaller angles than NiTi wires of the same cross section. This is due to the difference in elastic modulus of the different materials and to the super-elastic behaviour of NiTi. This is in line with Huang's simulations [17] using the finite elements approach.

In our study, the Nexus bracket was that with the highest engagement angles with respect to the experimental mean. This can be ascribed both to its oversize slot and the marked divergence in the slot walls. Furthermore, the NiTi clip of the active self-ligating Nexus may have undergone plastic deformation, thereby reducing its grip. This is an issue of clinical relevance, as clip reliability needs to be guaranteed, particularly in the finishing stages of orthodontic treatment. It is also possible that the brackets were subject to wear, being tested numerous times [22,23]. In their recent study, Major et al. [24] found that the plastic deformation of the slot is negligible when the applied torque is inferior to 26 to 38 Nmm. As in our study, it never exceeded 20 Nmm, it is conceivable that a possible plastic deformation occurred at the level of the clip and did not involve the metal walls, and it could explain the difference in play and torque expression between the Nexus and the other brackets.

Overall, the tests performed showed a high level of repeatability, although in certain cases a high standard deviation was detected, in all likelihood due to the small variations in size and edge bevelling between different samples of the same archwire, not to mention small variations in torquing key positioning.

It should also be noted that the test performed is only a simplified representation of that which occurs in the oral cavity. Indeed, only single brackets were tested without any tipping information. This is obviously not the case in real-life orthodontics, in which a multibracket appliance, composed of 12 to 14 brackets, will deflect an archwire in a far more complex fashion. Indeed, second-order misalignment will have an influence on the torque expressed, by increasing the friction between the archwire and the bracket [25]. Furthermore, the experiment was not designed to take into account the phenomenon of bracket wear, which can increase the slot height by as much as 0.02 mm [26]. Nevertheless, our findings do provide food for thought as regards the real expression of information by pre-angled appliances.

## Conclusions

The null hypothesis is rejected. In real life, the play between the archwire and the bracket slot is greater than the ideal, and the actual torque expressed by both 0.018 and 0.022 brackets will therefore always be less than expected. What is more, even when using brackets and archwires of nominally identical dimensions, there may be great variation in play and torque angles, due to the dimensional imprecision of such products. Unfortunately, it appears impossible to compensate for such discrepancies in a clinical setting, as, although in our experience, the bracket slots were invariably larger than their nominal size, some archwires were oversized and some were smaller than the measurements declared by the manufacturer, and the variable degree of bevelling will also affect the archwire/slot play.

## References

[1] Meling T, Ødegaard J (1998). The effect of cross sectional dimensional variations of square and rectangular chrome-cobalt archwires in torsion. Angle Orthod.

[2] Burstone CJ (1982). The segmented arch approach to space closure. Am J Orthod.

[3] Wahl N (2005). Orthodontics in 3 millennia. Chapter 2: entering the modern era. Am J Orthod Dentofacial Orthop.

[4] Wahl N (2008). Orthodontics in 3 millennia. Chapter 16: late 20th-century fixed appliances. Am J Orthod Dentofacial Orthop.

[5] Rinchuse DJ, Rinchuse DJ, Kapur-Wadhwab R (2007). Orthodontic appliance design. Am J Orthod Dentofacial Orthop.

[6] Kusy RP (1997). A review of contemporary archwires: their proprieties and characteristics. Angle Orthod.

[7] Kusy RP, Whitley JQ (2007). Thermal and mechanical characteristics of stainless steel, titanium-molybdenum, and nickel-titanium archwires. Am J Orthod Dentofacial Orthop.

[8] Johnson E (2003). Relative stiffness of beta titanium archwires. Angle Orthod.

[9] Gurgel JA, Pinzan-Vercelino CRM, Powers JM (2011). Mechanical properties of beta-titanium wires. Angle Orthod.

[10] Sifakakis I, Pandis N, Makou M, Eliades T, Katsaros C, Bourauel C (2013). Torque expression of 0.018 and 0.022 inch conventional brackets. Eur J Orthod.

[11] Detterline DA, Isikbay SC, Brizendine EJ, Kula KS (2010). Clinical outcomes of 0.018-inch and 0.022-inch bracket slot using the ABO objective grading system. Angle Orthod.

[12] Meling TR, Ødegaard J (1998). The effect of second-order couple on the application of torque. Am J Orthod Dentofacial Orthop.

[13] Meling TR, Ødegaard J (1998). On the variability of cross-sectional dimensions and torsional properties of rectangular nickel-titanium arch wires. Am J Orthod Dentofacial Orthop.

[14] Cash AC, Good SA, Curtis RV, McDonald F (2004). An evaluation of slot size in orthodontic brackets—are standards as expected?. Angle Orthod.

[15] Demling A, Dittmer MP, Schwestka-Polly R (2009). Comparative analysis of slot dimension in lingual bracket systems. Head Face Med.

[16] Rucker BK, Kusy RP (2002). Elastic flexural properties of multistranded stainless steel versus conventional nickel titanium archwires. Angle Orthod.

[17] Huang Y, Keilig L, Rahimi A, Reimann S, Eliades T, Jager A, Bourauel C (2009). Numeric modeling of torque capabilities of self-ligating and conventional brackets. Am J Orthod Dentofacial Orthop.

[18] Badawi HM, Toogood RW, Carey JPR, Heo G, Major PW (2008). Torque expression of self-ligating brackets. Am J Orthod Dentofacial Orthop.

[19] Flores DA, Choi LK, Caruso JM, Tomlinson JL, Scott GE, Jeiroudi MT (1994). Deformation of metal brackets: a comparative study. Angle Orthod.

[20] Flores DA, Caruso JM, Scott GE, Jeiroudi MT (1990). The fracture strength of ceramic brackets: a comparative study. Angle Orthod.

[21] Archambault A, Lacoursierea R, Badawi H, Major PW, Carey J, Flores-Mir C (2010). Torque expression in stainless steel orthodontic brackets. Angle Orthod.

[22] Kapur R, Sinh PK, Nanda RS (1999). Comparison of load transmission and bracket deformation between titanium and stainless steel brackets. Am J Orthod Dentofacial Orthop.

[23] Major TW, Carey JP, Nobes DS, Heo G, Major PW (2012). Deformation and warping of the bracket slot in select self-ligating orthodontic brackets due to an applied third order torque. J Orthod.

[24] Major TW, Carey JP, Nobes DS, Heo G, Melenka GW, Major PW (2013). An investigation into the mechanical characteristics of select self-ligated brackets at a series of clinically relevant maximum torquing angles: loading and unloading curves and bracket deformation. Eur J Orthod.

[25] Kang B, Baek S, Mah J, Yang W (2003). Three-dimensional relationship between the critical contact angle and the torque angle. Am J Orthod Dentofacial Orthop.

[26] Lacoursiere RA, Nobes DS, Homeniuk DL, Carey JP, Badawi HH, Major PW (2010). Measurement of orthodontic bracket tie wing elastic and plastic deformation by arch wire torque expression utilizing an optical image correlation technique. J Dent Biomech.

